# A method of diamorphine (heroin) administration for harm reduction

**DOI:** 10.1186/s12954-023-00758-1

**Published:** 2023-03-30

**Authors:** Oisín N. Kavanagh, Tatiane C. Machado

**Affiliations:** 1 School of Pharmacy, Newcastle University, Newcastle upon Tyne, UK; 2 Faculty of Medical Sciences, Translational and Clinical Research Institute, Newcastle University, Newcastle upon Tyne, UK

**Keywords:** Harm reduction, Heroin, Diamorphine, Phlebitis, Needle exchange programme, Citric acid, Ascorbic acid

## Abstract

As societal attitudes toward narcotics have changed, harm reduction strategies have emerged which make it safer to inject intravenous drugs. Diamorphine (heroin) is commonly sold as its free base—better known as brown—which has extremely poor aqueous solubility. As such, it needs to be chemically modified (cooked) to enable administration. Needle exchange programmes commonly supply citric or ascorbic acids which facilitate intravenous administration by increasing heroin solubility. If heroin users mistakenly add too much acid, the low solution pH can cause damage to their veins and, after repeated injury, could result in the loss of that injection site. Currently, advice cards supplied with these exchange kits suggest that the acid should be measured in pinches, which could result in considerable error. This work employs Henderson–Hasselbalch models to analyse the risk of venous damage by placing solution pH within the context of the buffer capacity of the blood. These models also highlight the significant risk of heroin supersaturation and precipitation within the vein, an event that has the potential to cause further harm to the user. This perspective closes with a modified administration method which could be included as part of a wider harm reduction package.

**Supplementary Information:**

The online version contains supplementary material available at 10.1186/s12954-023-00758-1.

## Background

Diamorphine (heroin) consumption can have negative impacts on the individual and society [1]. Much of these individual harms arise from unsafe injecting practices and initiatives such as needle exchange programmes (NEPs) have emerged in this context [2–5] to supply clean needles and other paraphernalia to drug users. Their success as a highly cost-effective strategy to reduce Hepatitis C transmission [6, 7] has led to legal changes which have widened access to this service in some countries. These kits often contain a tourniquet, clean needles and syringes, water for injection and citric or ascorbic acid.

While the former items are more obvious, supplying acids in these kits facilitates the dissolution of poorly water-soluble heroin that is found in the street (brown heroin). Although one cannot assume the purity of street heroin [8], this substance is effectively heroin base and residual plant material which needs to be dissolved in the injection solution to enable administration; injecting undissolved heroin would block the syringe or may directly damage the vein.

**Fig. 1:**
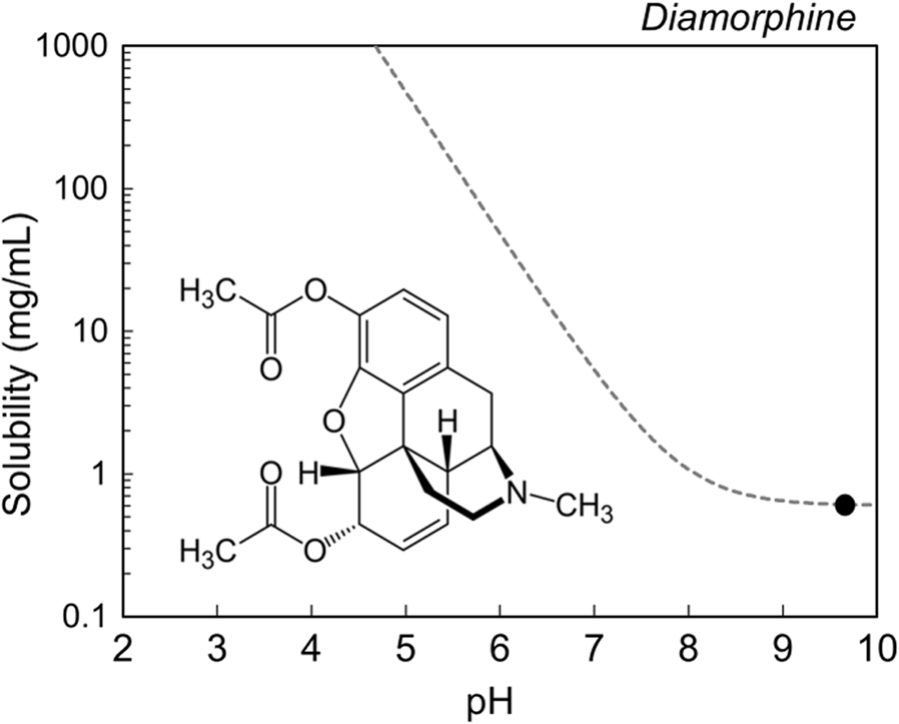
Diamorphine (Heroin) solubility curve (grey dashed line) as a function of pH modeled using Henderson–Hasselbalch theory. Experimentally determined solubility of heroin free base is indicated as a black dot [9] and its solubility will exponentially increase as pH decreases beyond its p*K*_*a*_. Diamorphine p*K*_*a*_ = 7.9 [11]

Heroin is a sparingly water-soluble compound (0.6 mg/mL) [9] which demonstrates increased solubility in an acidic, low pH environment (Fig. 1). Citric and ascorbic acids release hydrogen ions (H^+^) into solution, lowering the pH. The nitrogen containing morphinan group in heroin will react with these H^+^, enabling the heroin to ionize and ultimately dissolve in water. White pharmaceutical grade heroin—which is also sold illegally—is the heroin free base combined with an acid such as hydrochloric acid (HCl) which makes a salt (known as white heroin).

$${\text{Heroin}} + {\text{H}}^{ + } \leftrightarrow {\text{Heroin-}} {\text{H}}^{ + }$$

This salt (diamorphine hydrochloride) is 1000 times more soluble than heroin free base and does not need pH adjustment as it will readily dissolve in water (600 mg/mL) [9]. Heroin salts such as Heroin·HCl or Heroin·Citrate and Heroin·Ascorbate will self-buffer the solution to a pH at which the active compound is soluble. Solubility in this context relates to the ability of a typical dose to dissolve in an appropriate volume to facilitate intravenous delivery. For example, a typical heroin dose is around 100 mg (± 50 mg) [10], and when dissolved in 1 mL, requires a pH of approximately 5.5 to fully dissolve the dose. Figure 1 illustrates the solubility profile of heroin across a range of pH values.

There are many methods of preparation or cooking heroin with acids such as ascorbic and citric acid, and some methods are more harmful than others [12, 13]. Problems arise when users attempt to add too much acid to dissolve the heroin base, this results in an acidic solution which can cause phlebitis, inflammation of the peripheral veins. It is likely that peripheral veins can withstand a pH shift of up to one unit from physiological values of 7.4 (i.e., 6.4–8.4) and phlebitis reactions will occur at pH extremes beyond this [14, 15]. Current information supplied with citric and ascorbic acid sachets in NEPs recommend that pinches of acid should be added to a suspension containing heroin. Clearly, this can lead to considerable error, and if excess acid is used, can produce a highly acidic solution which is likely to cause damage [16, 17]. Manufacturers of citric and ascorbic acids (Exchange supplies, UK) have reduced the amount of acid from 200 to 100 mg per sachet to prevent this and some authors have advocated for the use of ascorbic acid to reduce the risk of harm [18], but even this will create solutions whose pH will cause significant irritation to the injection site.

This work addresses three aspects of heroin administration to improve safety: (1) What is the minimum quantity of acid required to dissolve heroin? (2) How do we instruct users to add this small quantity of acid? (3) How quickly should users inject this infusion?

## Finding the minimal quantity of acid

**Fig. 2:**
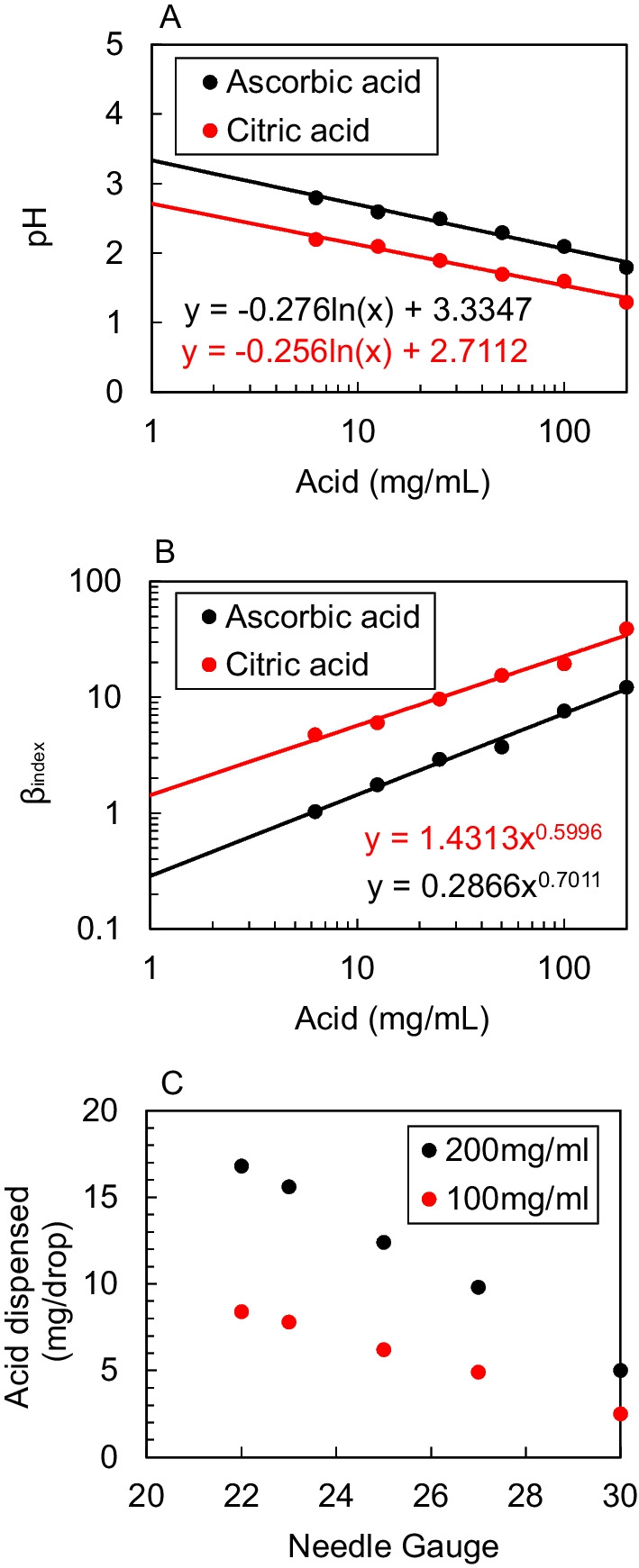
**A** Solution pH of ascorbic or citric acid in saline. **B** *β*_index_ for a 5 s infusion rate for ascorbic or citric acid mixed with a 100 mg dose of heroin base. The *β*_index_ is a ratio of the excess mEq of [H^+^] added by the acid and the buffer capacity of the blood. *β*_index_ > 1 suggests the induction of a clinically relevant pH shift (> 1 pH unit, increasing the risk of phlebitis). **C** Small needle gauges can enable small quantities of acid to be aliquoted dropwise, adapted from Tripp et al. [24]

The addition of an acid or base to a solution can establish an equilibrium by adding or removing free hydrogen ions ([H^+^]) from it and this equilibrium can be described by the dissociation constant *K*_*a*_. Ascorbic acid is known to be less acidic than citric acid and we find that concentrations from 200 to 6.25 mg/mL led to pH values in the range of 1.8–2.8 (ascorbic acid) and of 1.3–2.2 (citric acid) (Fig. 2A). However, the ideal infusion solution will have just enough acid to completely react with heroin, without excess. Adding acid to a solution containing heroin base will create a salt which will buffer the solution around a certain pH value. The pH of a buffer solution can be estimated using the dissociation constants of the acid (citric or ascorbic) and base (heroin) present in solution [19] (Eq. 1).

1 $${\text{pH}} = 7 + \frac{1}{2}\left( {pK_{{a, {\text{acid}}}} - pK_{{a,{\text{heroin}}}} } \right)$$

This equation is simplified as we assume that ionic strength is > 0.01 M and constant, therefore activity is constant. Although citric acid is polyprotic (it has potentially three [H^+^] which can be released into solution), we can consider just the first deprotonation event as *K*_*a*1_ >  > *K*_*a*2_ >  > *K*_*a*3_. Further, we assume that as heroin has a p*K*_*a*_ > 2 units beyond citric and ascorbic acid (p*K*_*a*, ascorbic acid_ = 4.7 vs. p*K*_*a*, citric acid_ = 3.1), we would expect almost all (> 99%) of the heroin to react with the available hydrogen ions ([H^+^]) donated by the acid, such that we can calculate the pH of a solution of equimolar acid and heroin [19]. As such, the optimal dose of citric and ascorbic acid to dissolve a typical 100 mg dose of pure heroin (0.00027 mol, p*K*_*a*_ = 7.9) in 1 mL is 5.1 and 4.7 mg and should generate a solution of pH 4.6 and 5.4, respectively. Other authors have revealed that 97.46 mg heroin dissolved in 0.7 mL deionized water containing 20 mg citric acid produces a solution of pH 2.55 [18, 20]. Our calculations suggest that this quantity of heroin and citric acid should produce a solution of pH 1.8 (Fig. 2A). Experimental data from Scott suggests that it requires more citric and ascorbic acid (in excess of 30 and 60 mg, respectively) [20] than expected to dissolve a heroin dose. We assume that these discrepancies are due to the presence of contaminants.

Although pH 4.6 and 5.4 are significantly beyond the pH of the blood (i.e., > 1 pH unit from 7.4), the blood has a mechanism of acid-basic regulation that can compensate for significant pH changes that may occur, known as the buffer capacity. The buffer capacity of blood is expressed in mEq/L/pH and describes the quantity of acid or base required to cause a pH shift of 1 unit in a 1 L solution [21, 22]. Based on this knowledge of blood buffer capacity and Henderson Hasselbalch concepts, we propose an index—the buffer index, *β*_index_—which describes a heroin infusion pH that can overcome this buffering capacity. The *β*_index_ is calculated from the ratio of excess hydrogen ion concentration [H^+^] and the buffer capacity of the blood (*β*), in the context of in the peripheral vein blood flow rate (210 mL/min) [23] and the administration time, which we have assumed to be 5 s ($\beta^{*}$), Eqs. 2 and 3. Evidence suggests that the peripheral vein can withstand pH changes of up to pH 6.4 [14, 15]. Therefore, *β*_index_ values > 1 (i.e., a pH shift > 1 unit from 7.4) may increase the risk of causing phlebitis at the injection site.

2 $$\beta^{*} = { }\beta \left( {\frac{{\frac{{\text{M}}}{{{\text{mL}}}}}}{{{\text{pH}}}}} \right) \times {\text{ Blood}}\;{\text{flow}} \left( {\frac{{{\text{mL}}}}{{\text{s}}}} \right) \times {\text{Administration}}\;{\text{time}} \left( s \right)$$

3 $$\beta_{{{\text{index}}}} { } = { }\frac{{[{\text{H}}^{ + } ] - \left[ {{\text{Base}}} \right]}}{{\beta^{*} }}{ }$$

When these acidity values are placed in the context of a blood buffer capacity of 73 mEq/L/pH [21, 22] and flow rate of 210 mL/min [23] (Fig. 2B), we reveal that for pure heroin, > 0.7 mg citric acid and > 6 mg ascorbic acid would exceed the *β*_index_ of 1, potentially causing phlebitis. Although we know that these calculations underestimate the amount of acid required to produce β_index_ > 1—due to the presence of contaminants—we suggest that because of the small margins of error, ascorbic acid should be preferred over citric acid.

## How to add milligram amounts of acid

Another problem which arises as users attempt to add this small quantity of acid to prevent the dose from exceeding the *β*_index_. In interviews conducted with heroin users Scott reports, “…*about 10 grains… or a small pinch*” [20] is a typical dose of acid, and one pinch is around 15 mg [18]. Accurate and precise measurements of < 0.1 g by this method are highly unreliable. It should be noted that various batches of heroin may contain different quantities of impurities, and as such, attempting to completely dissolve all solids may invertedly over-acidify the dose without increasing the concentration of heroin.

We illustrate in Fig. 2C how greater accuracy and precision can be achieved using the needle and syringe supplied in the NEP by creating a solution of 100 mg/mL acid and adding it dropwise. Then, we propose that drug users should be advised to dissolve the entire 100 mg pack of acid into a fixed volume of water, e.g., 1 mL, as per the maximum volume of a typical syringe in a NEP pack. In a 100 mg/mL solution, each drop will contain < 5 mg (Fig. 2C). Users can then add the acid dropwise to a heated suspension of heroin and water (approximately 0.5 mL) until heroin dissolution is observed. Then users can discard the remainder of the acid in the syringe, top-up the solution with water and draw the dose up into the same syringe.

## How long should administration take?

**Fig. 3:**
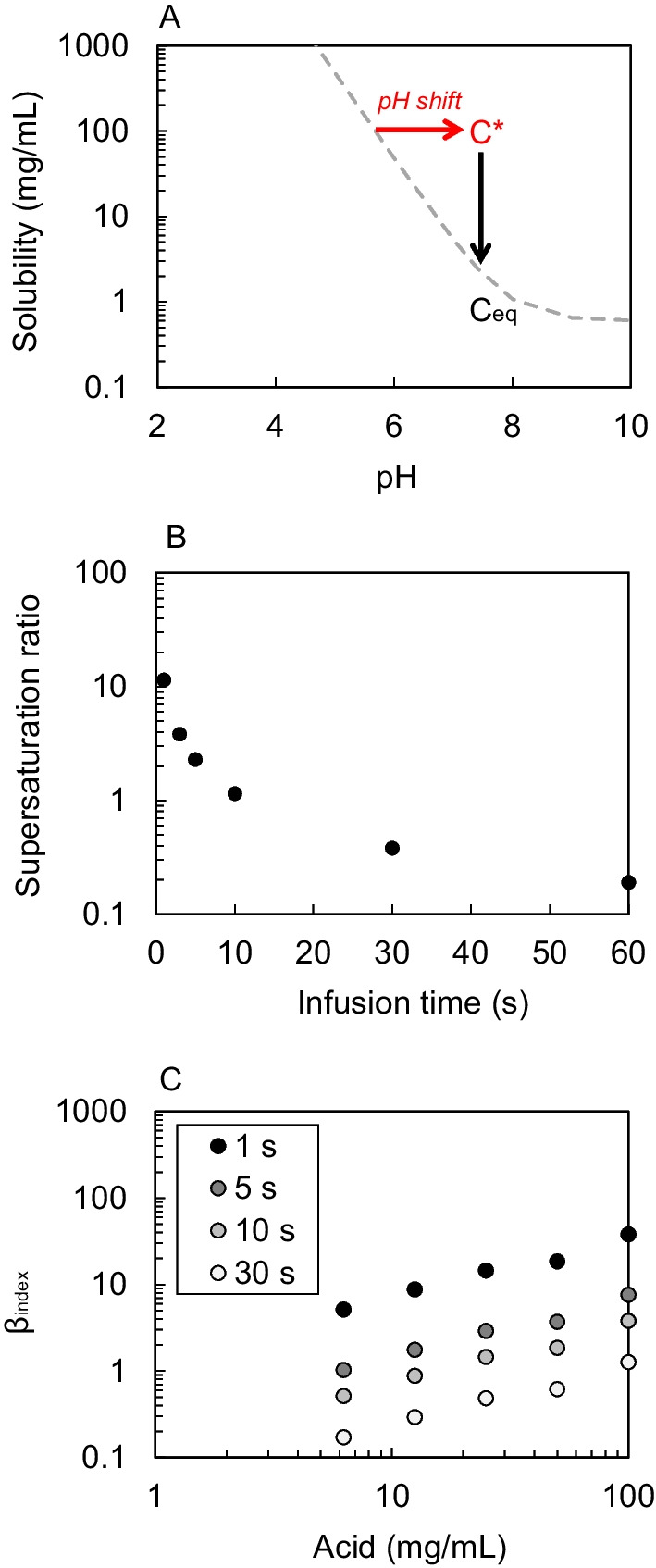
**A** A typical heroin infusion will generate supersaturation as the heroin concentration (C*) is higher than the heroin solubility (*C*_eq_) at blood pH 7.4. The pH shift and precipitation processes are indicated by the arrows. **B** The rate of infusion on the generated supersaturation ratio, slower rates provide more time for a greater blood volume, effectively diluting the solution and reducing the supersaturation ratio. Larger supersaturation ratios will result in a more rapid rate of precipitation. **C** Increasing infusion time can significantly reduce the *β*_index_, as illustrated using ascorbic acid data

One aspect of heroin administration that has not been addressed is the potential to generate supersaturation by infusing a heroin solution of pH 5.0 (the pH value required to dissolve a typical 100 mg heroin dose) into the vein, which is buffered to pH 7.4 (Fig. 3A). Supersaturation describes a state where the concentration of a substance in solution at a particular moment (C*) is above its equilibrium state (*C*_eq_) (Eq. 4). As the supersaturation ratio describes how far away a solution is from its equilibrium state, a supersaturation value > 1 indicates a risk of precipitation, higher values indicate higher precipitation rates and therefore greater risks of precipitation.

4 $${\text{Supersaturation}}\;{\text{ratio}} \left( \sigma \right) = \frac{{C^{*} }}{{C_{{{\text{eq}}}} }}$$

These circumstances can be induced by a pH shift whereby the prepared heroin solution—at pH around 5 to enable heroin dissolution—will be shifted toward pH 7.4. At this pH, the heroin in solution will be above its equilibrium state (*ca.* 2–3 mg/mL). Precipitation (i.e., removal of the drug from solution) will then occur to restore the drug concentration in solution to equilibrium (Fig. 3A). Equation 5 and Fig. 3B place the heroin solubility in the context of blood flow and the rate of infusion to describe a physiologically relevant supersaturation ratio.

5 $${\text{Supersaturation}}\;{\text{ratio}} \left( \sigma \right) = \frac{{\left( {\frac{{{\text{Heroin}}\;{\text{dose}}}}{{{\text{Blood}}\;{\text{flow}} \times {\text{ rate}}\;{\text{of}}\;{\text{infusion}}}}} \right)}}{{{\text{Solubility}}\;{\text{at}}\;{\text{pH}}\;7.4 }}$$

For example, the supersaturation ratio for a typical dose of heroin (100 mg/mL) and its solubility at pH 7.4 (2 mg/mL) infused within 1 s (blood volume = 3.5 mL) is > 10. This is very likely to result in rapid precipitation of heroin in the vein. Figure 3B shows that although the risk could be minimized with an infusion time of 10 s or longer, it is possible that users may not comply with this recommendation as it may diminish the high. We recognize that although greater volumes could also be used to administer heroin, providing another means to minimize risk, this may result in a diminished high, reducing compliance. Conversely, highly concentrated heroin infusions will increase the risks of supersaturation and so we recommend that heroin concentrations ≤ 100 mg/mL should be administered. Increasing infusion times in this way has the added benefit of decreasing the *β*_index_ < 1 (Fig. 3C). Calculations for these models are available in the supplementary text (Additional file 1).

## Conclusions

We bring the concepts of the Henderson–Hasselbalch equation to the context of blood buffer capacity to propose an index (*β*_index_) that provides a means to reduce the risk of bolus intravenous drug administration, particularly where phlebitis is a risk. The approach proposed could be applied to other intravenous therapies such as the benzodiazepines, diazepam and lorazepam or Iron infusions (Jectofer®) and will be explored in these contexts in future work.

This accurate, safer infusion method should be advocated for heroin users and could be provided by the pharmacist or harm prevention work within their NEP. The information provided on the card within the kit should also be amended to reflect these findings. The accuracy and precision offered by this new approach could feasibly be carried out by a user and requires less manual dexterity than the previously recommended pinch approach [18]. This could lead to harm reduction for many patients who inject drugs.

**Table:** 

Proposed method *Dissolution of ascorbic acid* 1. If using premade packets, tip the entire 100 mg packet into a sterile cooker. 2. Dissolve the entire packet of acid in at least 1 mL of sterile water. 3. Withdraw the entire solution into the syringe and use the same cooker for the next step. *Preparation of heroin dose* 1. Add heroin to a sterile cooker and add around 0.5 mL sterile water to make a suspension. 2. Hold the acid loaded needle at 90 º to the cooker. 3. Heat gently and add the acid solution until the heroin dissolves. *Add 0.1 mL to start and then dropwise.* 4. Draw dose into syringe (through filter if required). 5. Inject the heroin solution slowly, taking more than 5 s. *Use a timer on your phone to get it right*. 6. If tourniquet is required, remember to release prior to infusion. 7. Discard the remainder of the acid.	

We appreciate that this method may not be practical for those who inject opioids in unsafe spaces and may not have the time to undertake this method.

## Supplementary Information

**Additional file 1.** Excel file containing calculations for figures. Sheet 2 contains calculations relating to the pH of a solution containing various quantities of acid. These calculations are then contextualised using the B-index for solutions containing heroin. Sheet three contains data extracted from Tripp et al. on the mass of acid delivered per drop (assuming 100 mg/mL concentration). Sheet 4 contains calculations of heroin solubility as a function of pH.

## Data Availability

All data are available in the Additional file 1.
